# Draft genome sequence of a *Klebsiella pneumoniae* virulent bacteriophage vB_KpBD_211 isolated from wastewater in Dhaka, Bangladesh

**DOI:** 10.1128/mra.00909-25

**Published:** 2025-12-23

**Authors:** Mst. Morioum Sarkar, Tasnimul Arabi Anik, Sangita Ahmed

**Affiliations:** 1 Department of Microbiology, University of Dhaka 95324, Dhaka, Bangladesh; Portland State University, Portland, Oregon, USA

**Keywords:** bacteriophage genetics

## Abstract

*Klebsiella pneumoniae* phage vB_KpBD_211, isolated from wastewater in Dhaka, Bangladesh, has a 47,938 bp double-stranded DNA genome (57.05% GC), assembled from Illumina MiSeq data, and lacks virulence or antibiotic resistance genes, supporting its potential in MDR *K. pneumoniae* therapy.

## ANNOUNCEMENT

**Fig 1:**
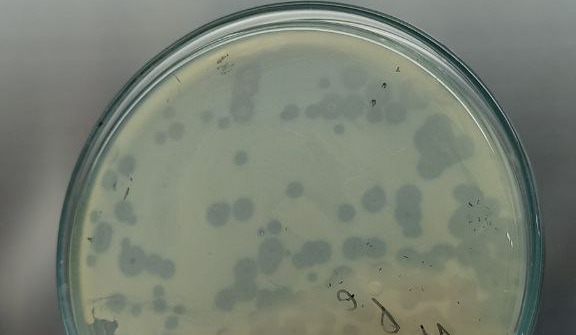
Plaques formed by phage Bacteriophage vB_KpBD_211 on *Klebsiella pneumoniae* strain CP4 lawns using the double-layer agar method.

*Klebsiella pneumoniae* is a Gram-negative pathogen associated with multidrug resistance and high virulence (1, 2). Virulent bacteriophages offer a promising alternative to antibiotics for controllinog MDR *K. pneumoniae* infections (3). To explore phage-based strategies against MDR *K. pneumoniae*, we isolated a virulent phage, vB_KpBD_211, from wastewater of a sewage treatment plant (WASA, Dhaka) on 12 October 2024 (Fig. 1). Samples were centrifuged and sequentially filtered through a 0.22-µm membrane filter. The phage was isolated by adding 100 µL of the filtered sewage to 100 µL of a multidrug-resistant *K. pneumoniae* strain (CP4) in logarithmic phase (~10⁸ CFU/mL) using the double-layer agar assay, and the incubation was performed at 37℃ for 24 h (4). A purified plaque (3–4 mm) was amplified through three successive passages. Lysates were prepared on LB agar by overlaying confluent lawns and flooding with SM buffer after plaques formed (12–18 h); collected buffer was centrifuged (10,000 × *g*, 10 min), filtered (0.22 µm), and used to prepare high-titer stocks (~10^9^ PFU/mL) for DNA extraction. Lysates were treated with DNase I/RNase I (80 µg/mL) at 37 °C for 3 h prior to PEG precipitation and DNA extraction using a modified protocol of the DNeasy Blood and Tissue Kit (Qiagen) (5).

Library preparation was performed using the Illumina DNA Prep kit (Illumina, San Diego, CA, USA), and sequencing was performed on the Illumina MiSeq platform. A total of 187,404 raw reads (paired) were generated, corresponding to 45.2 Mb of sequencing data (read length 250 bp). The sequenced genome had an average depth of coverage of 698.76×. In the sequence analysis, default parameters were used. Raw reads were quality-checked using FastQC (v0.12) (6) and trimmed with Trimmomatic (v0.39) (7). *De novo* assembly was carried out using SPAdes (v4.1) (8), and assembly quality was evaluated using QUAST v5.3.0 (9). Genome annotation was performed with Pharokka v1.7.5 (10). Genome completeness and quality were assessed using CheckV v1.0.1 (11). The taxonomic relationships of the phage were determined by NCBI BLASTn (12). The virulence factors and antibiotic resistance factors were detected using the VFDB v2.00 (13), and ResFinder v4.00 (14) database, respectively.

The genome of phage vB_KpBD_211 is a 47,938 bp linear double-stranded DNA (dsDNA) with 57.05% GC content, 96.77% completeness, and 0.00% contamination (Table 1). Linearity was inferred from the assembly structure visualized in Proksee (15). The phage vB_KpBD_211 is a new species assigned to the family *Drexlerviridae*, genus *Webervirus,* as determined by the TaxMyPhage tool (16). CheckV predicted 68 viral genes and confirmed the absence of host-derived sequences in the phage genome. Several genes encoding proteins involved in bacterial cell lysis were identified, including endolysin, holin, and spanin. *Klebsiella* phage RAD2 (Reference sequence accession number GCF_020405405.1) is the closest relative to vB_KpBD_211, with annotated proteins, such as endolysin, holin, and tape measure protein shared 97.06%, 98.59%, and 95.75% amino acid identity, respectively, after alignment with BLASTp. The phage vB_KpBD_211 contains no virulence, antibiotic-resistant genes, and genes responsible for lysogeny. This genome adds to the growing repository of virulent *Klebsiella* phages for therapeutic use.

**TABLE 1:** General features of the assembled whole genome of *Klebsiella pneumoniae* virulent bacteriophage vB_KpBD_211

Features	*Klebsiella pneumoniae* virulent bacteriophage vB_KpBD_211
Phage genome length (bp)	47938
Coverage	698.76
GC content (%)	57.05
Completeness (CheckV)	96.77%
Contamination (CheckV)	0.00%
Open reading frames (ORFs) (predicted by Pharokka)	81
Hypothetical proteins (predicted by Pharokka)	47
Viral genes (CheckV)	68
Quality (CheckV)	High
Host gene (CheckV)	Absent
Life cycle	Lytic
Virulence gene	No
Antibiotic resistance gene	No

## Data Availability

The raw reads can be found at NCBI Sequence Read Archive (SRA), accession number SRX2956653. The assembly of raw reads is available at NCBI BioProject, accession number PRJNA1287049 and BioSample accession number SAMN49806216. The viral genome sequence is available in GenBank under accession number PV893054. Host genome accession number GCF_039872675.1.
